# Long-Term Follow-Up of Gender-Affirming Chest Masculinization: What Have We Learned About Patient Satisfaction and Psychological Well-Being?

**DOI:** 10.3390/jcm14041249

**Published:** 2025-02-13

**Authors:** Samuel Kwartin, Ron Skorochod, Liran Shapira, Yoram Wolf

**Affiliations:** 1 Department of Plastic and Reconstructive Surgery, Hillel Yaffe Medical Center, Hadera 3820302, Israelron.skorochod@mai.huji.ac.il (R.S.);; 2 Technion-Institute of Technology, Rappaport Faculty of Medicine, Haifa 3525433, Israel

**Keywords:** Gender affirmation, plastic surgery, gender affirming mastectomy, PROM

## Abstract

**Background:** Gender-affirming surgery has become an integral part of the gender transition process that transgender and gender-diverse individuals undergo. Although ample literature exists on the short-term outcomes of gender-affirming surgery, very little is known about the long-term implications the surgery has on the psychological well-being of the patients. The purpose was to understand the long-term impact that gender-affirming surgery has on transgender and gender-diverse individuals and gain insight on potential contributors to improved psychological well-being and satisfaction. **Methods:** All patients who were operated on by a single surgeon during a 20-year period were invited to the clinic for a follow-up appointment. The patients were physically examined, their scars were graded, and NAC sensation was evaluated. BUT (A and B) and BREAST-Q questionnaires were filled out by them and evaluated by the research staff. **Results:** Satisfaction with pre-operative information provided to the patient was associated with satisfaction with the final appearance of the chest (R = 0.717, *p* < 0.001), the surgical outcome (R = 0.481, *p* = 0.037), psychosocial well-being at follow up (R = 0.489, *p* = 0.034), satisfaction with the surgeon (R = 0.486, *p* = 0.035), satisfaction with the medical team (R = 0.62, *p* = 0.005) and satisfaction with the office staff (R = 0.65, *p* = 0.003). **Conclusions:** Pre-operative communication between the medical staff and the patients improves the psychological outcomes and satisfaction of the patients over the years.

## 1. Introduction

Gender dysphoria is defined as the mental distress associated with a mismatch between the individual’s gender identity and the sex with which that person was born. The mainstay of treatment clinicians and mental health specialists have to offer consists of counseling, hormonal therapy and surgical modalities [1,2].

Over the last decades, we have seen a significant increase in the number of people who identify as transgender or gender-diverse (TGD) and, subsequently, an increase in the number of people expressing interest in gender-affirming care [3].

Although hormonal therapy is typically the first step in the gender-affirmation process, surgical procedures play a vital role in alleviating the feeling of gender dysphoria [4]. Surgical mastectomy is commonly the first surgical intervention that transgender males pursue in their transition process, and in many cases, it can also be the only intervention [5].

There are ample techniques and modifications to the traditional mastectomy to better suit the needs and desires of TGD patients. The surgical technique is tailored to the specific patients and takes into consideration comorbidities, breast size and the patient’s desires in terms of NAC (Nipple-areolar complex) for achieving optimal results [6,7,8].

The configuration of the male breast differs from the female breast, not only in volume and shape but also in the size and position of the nipple-areolar complex. When the female breast is small or more androgenic in appearance, a circumareolar approach can produce a satisfactory result. Patients with macromastia or ptotic breasts require large incisions, which commonly include transverse incisions in the inframammary fold (IMF) to completely resect the entire glandular tissue. 

Traditionally, the pre-determined location of the nipple-areolar complex (NAC) area is set using the free nipple graft method [9,10,11]. As an alternative to the free nipple graft technique, it is possible to operate with a method that uses excess skin as a pedicle for the NAC and later allows fixation of the NAC in its new position without the need to use a nipple graft. This method is known as the inferior pedicle method and is believed to better preserve the structure of the NAC [12,13].

In a previous study that compared the sensation after mastectomy surgery with the two methods among women who underwent breast reduction surgery, the authors witnessed a return of sensation after both surgeries, but the transgender population who underwent the surgery as part of gender reassignment medical treatment was not examined [14].

The aesthetic outcome and the appearance of the scars play a vital role in the patients’ satisfaction with the outcome. Sub-par healing of the operated chest results in a perceived impairment of the outcome and incomplete resolution of gender-dysphoria feelings [15,16].

In this study, we aim to analyze the long-term patient satisfaction and surgical results after gender-affirming mastectomies. Several methods will be employed to portray a detailed picture of the patient’s well-being in the long-term analysis of the results.

## 2. Materials and Methods

### 2.1. Patient Recruitment

The study presented herein is a retrospective cohort study of all TGD patients operated on by the senior author during the years 2003–2019. All surgical mastectomy patients who agreed to return to the clinic for the clinical and psychological assessment were included in this study. Patients were excluded from the study if they did not fulfill the inclusion criteria and in instances when the patient was a minor at the time of the observation conducted in this study.

### 2.2. Data Extraction

The medical records and surgical reports of all included patients were assessed and analyzed. Relevant demographical, clinical and surgical data was extracted. All included patients were invited for a follow-up appointment that involved the completion of an additional review session. In this visit, the patients were asked to complete the BREAST-Q and Body-Uneasiness Test (BUT) A and B questionnaires [supplementary digital material 1–3]. The NAC sensitivity was assessed by comparing adjacent skin to the NAC using a 10 g monofilament test, a 128 Hz metal tuning fork was used for vibration and cold sensation and light touch was assessed with an applicator. In addition, two-point discrimination was assessed using a designated device. Surgical scars were graded using the Vancouver scar scale by two independent research coordinators.

### 2.3. Statistical Analysis

Descriptive statistics in terms of mean, standard deviation, percentages and ranges were calculated for all the parameters in the study. The relation between continues parameters was tested by Pearson correlation. *t*-test and Mann–Whitney U test were used to assess the differences between different groups. A linear regression model was used to predict Breast Q score parameters with adjustment to age at surgery and resection of breast weight. Paired tests were used to test the changes before vs. after the surgery for the body uneasiness test. A *p* value equal to or smaller than 0.05 was considered significant. SPSS version 28 was used for all statistical analyses.

## 3. Results

Contact information was available for 104 eligible patients, of which 43 could not be reached, 26 refused to participate and 15 did not arrive to the scheduled appointment. As a result, 20 patients were examined and included in the study.

The most reported reason for the low response rate to follow-up invitations was the change in names, home addresses and phone numbers that impaired the study coordinators’ ability to locate the patients.

The average age at the time of surgery was 20.9 (±4.4) years, with an average age of testosterone treatment initiation of 19.75 (±4.6) years. The mean time period between surgery and the final follow-up appointment was 99.85 (±64.2) months.

In regard to the surgical technique used, four patients (20%) were operated with a periarolar approach, six (30%) with the inferior pedicle flap and ten (50%) with free NAC flap (Table 1).

The analysis of the Breast Q questionnaire demonstrated that the average satisfaction with chest appearance and outcome scores were 89.7 ± 8.75 and 93.5 ± 8.1, respectively. The average psychosocial and sexual well-being scores were 78.8 ± 14.6 and 82.2 ± 10.5, respectively. The average score for satisfaction with the nipple-areolar complex was 77.2 ± 18.6, and those with the information given by the surgeon, the surgeon himself, the entire medical team and the office staff were 90.53 ± 12.26, 92.2 ± 13.38, 96.6 ± 8.9 and 93.3 ± 16.14, respectively (Table 2).

The results of the body uneasiness test (BUT A and BUT B) questionnaire demonstrated a statistically significant change in both questionnaires’ scores between initial pre-operative scores and the ones reported at the final follow-up appointment [Table 3].

Scar assessment using the Vancouver scar scale demonstrated an average score of 1.79 ± 0.98, elevated namely by the pigmentation and vascularity parameters. The nipple-areolar complex (NAC) was assessed for sensation using a 5-point Likert scale comparing normal chest skin with the NAC using a 10 g monofilament, a Q-tip for light touch and a 128 Hz metal tuning fork for vibration and cold sensation. In addition, 2-point discrimination was assessed using a designated device (Table 4).

Extensive follow-up was associated with decreased satisfaction with the surgical outcome (r = −0.473, *p* = 0.035). However, a negative correlation was observed between the follow-up time and the improvement of the BUT score (r = −0.487, *p* = 0.034) [Table 5].

Satisfaction with pre-operative information provided to the patient was associated with satisfaction with the final appearance of the chest (R = 0.717, *p* < 0.001) the surgical outcome (R = 0.481, *p* = 0.037), psychosocial well-being at follow-up (R = 0.489, *p* = 0.034), satisfaction with surgeon (R = 0.486, *p* = 0.035), satisfaction with medical team (R = 0.62, *p* = 0.005) and satisfaction with the office staff (R = 0.65, *p* = 0.003) (Table 6).

In terms of NAC sensation, no significant correlation was found between NAC sensation and BUT score improvement, type of surgery, breast classification nor the resection weight. However, increased NAC sensation was positively correlated with psychological well-being (r = 0.532, *p* = 0.019) (Table 7).

## 4. Discussion

Gender-affirming mastectomies are a turning point in the lives of transgender and gender-diverse individuals. They often represent the pivotal step in the gender-affirming journey, when the physical appearance matches the gender with which they identify. Beyond the physical transformation, gender-affirming procedures can have profound implications on the psychological state and quality of life of TGD individuals. Recognizing the importance of these outcomes is fundamental to providing comprehensive care and increasing patient satisfaction.

The current literature mainly focuses on immediate post-operative outcomes, with scarce resources dedicated to investigating the long-term implications of gender-affirming interventions on the physical and mental well-being of the patients. 

The advantage of assessing outcomes after substantial periods of time lies in the possibility of assessing the complete healing of the surgical site, the formation of scars and the formation of a physical appearance conforming to the gender-norm perceptions of third-party viewers. The results of such studies can provide valuable insights into patients at risk for regret, dissatisfaction with the outcome or need for revision surgery.

Recognition of the importance of patient-centered care in various surgical fields has driven researchers to identify patients at higher risk for post-operative complication rates, psychological distress and longer length of stay in the hospital setting. Researchers have concluded that an important contributor to optimal surgical results and rehabilitation in the general population of patients is thorough and comprehensive pre-operative patient education. Brodersen et al. [17] conducted a systematic literature review of studies evaluating the impact of pre-operative patient education on post-operative outcomes in abdominal surgery. The authors analyzed 12 studies and found that in most reports, pre-operative patient education resulted in a reduction in length of the hospital stay, post-operative complications or adverse events and psychological distress and anxiety surrounding the surgery.

Moreover, a review conducted by Powell et al. [18] found that psychological preparation before general and aesthetic elective surgery can be beneficial in reducing post-operative pain, behavioral recovery, negative affect and length of stay.

Similarly, the statistical analysis of the results presented in our study demonstrates conclusively that satisfaction with the pre-operative information and with the surgical and paramedical team are directly correlated with greater overall satisfaction with the surgical result and psychological well-being at substantial follow-up periods.

Overall satisfaction and psychological well-being, as studied in our research, was found to be additionally positively correlated with nipple-areolar complex (NAC) sensation. This finding should come as no surprise, as ample literature has suggested the importance of NAC sensation to patients undergoing breast surgery [19,20,21,22,23,24,25,26,27,28]. However, we did not find variables that were statistically significantly associated with decreased NAC sensation, including surgical technique, resection weight and pre-operative patient phenotypical characteristics.

In this context, it is crucial to address the potential confounding effect that the surgical technique has on the analysis of NAC sensitivity. In our study, we found no association between the surgical technique and greater NAC sensitivity. Yet, we believe it can serve as an effect moderator and should be taken into consideration.

Immediately, the findings of our study seem intuitive; it is easy to expect greater patient satisfaction when the pre-operative setting is tailored to ensure comfort and reassurance. However, the main purpose of our study was to reach out to patients a substantial period after surgery and identify key factors that were important to them. In other words, the uniqueness of our study stems from the focus on the patient perspective and analysis of their answers.

Even though this finding is based on a small sample size of patients, and conclusive evidence is still required to draw definite conclusions, it reinforces the importance of NAC sensation to patient well-being in long-term follow-up. Additionally, it suggests that no explicit factors have been proven to decrease NAC sensation in this cohort of patients.

Meaningful conclusions were obtained from the results of our study; however, its limitations must be taken into consideration. Namely, the small sample size and non-cohesive follow-up periods. Additionally, we recognize the potential for misclassification bias due to the low response rate in our survey-based research.

The choice to perform this study in a retrospective manner should also be raised as a potential limitation. Although retrospective analyses of results and patient outcomes allow for thorough analyses, they lack the possibility to establish causality. In this instance, we opted for a retrospective study to identify the association between patient and operator-related factors and long-term outcomes. Since it was impossible to predict future non-responders and patients lost to follow-up, we opted to assess the data retrospectively. Future studies should focus on prospective evaluation of outcomes to better discuss causality.

The aim of our study, as outlined throughout the manuscript, was to provide preliminary evidence of clinical and patient-reported outcomes of gender-affirming mastectomies at substantial follow-up periods to serve as an interim analysis. Despite the sample size being on the smaller side, the statistical significance of the results presented highlights an important point that we believe the medical community should be familiar with.

In conclusion, our study reinforces the importance of assessing long-term outcomes after gender-affirming mastectomies. In addition to the physical change patients experience in the post-operative period, profound psychological changes are expected to occur, and mitigation strategies aimed towards reducing the aspects that contribute to the psychological distress should be considered. Our findings suggest that preserving NAC sensation and ensuring effective communication in the pre-operative setting are critical components of optimal clinical and patient-reported outcomes. By recognizing and addressing these factors, healthcare providers can contribute to improved patient satisfaction, psychological well-being, and surgical results.

## Figures and Tables

**Table 1:** Patients’ demographics and surgical approach.

Age at surgery (years)	20.9 ± 4.4 (14–35)
Testosterone starting age (N = 16)	19.75 ± 4.6 (14–34)
Classification	
1—I	0
2—IIa	4 (22%)
3—IIb	8 (44%)
4—III	6 (33%)
Surgery	
1—Periareolar	4 (20%)
2—NAC * flap	6 (30%)
3—Free NAC	10 (50%)
Weight of resection left breast (grams)	317.5 ± 183
Weight of resection right breast (grams)	326 ± 175
Revision surgery	4 (20%)
Average time of follow-up (months)	99.85 ± 64.2 (45–232)

* NAC = Nipple-areolar complex.

**Table 2:** Breast-Q questionnaire score.

Satisfaction with Chest (11–44)	40.6 ± 2.89
Satisfaction with Outcome (5–15)	14.35 ± 0.81
Psychosocial Well-Being (9–45)	37.4 ± 5.25
Sexual Well-Being (6–30)	27.73 ± 2.52
Physical Well-Being (0–32)	26.55 ± 3.94
Satisfaction with NAC appearance (5–20)	16.58 ± 2.85
Satisfaction with Information (10–40)	37.16 ± 3.38
Satisfaction with Surgeon (12–48)	45.2 ± 4.82
Satisfaction with Medical Team (7–28)	27.3 ± 1.87
Satisfaction with Office Staff (7–28)	26.6 ± 3.39

**Table 3:** Body Uneasiness Test (BUT) A and B questionnaire score.

	Before (0–170)	Now (0–170)	
BUT A score	100.3 ± 36.75	41.47 ± 21.16	*p* < 0.001
BUT B score	45.32 ± 25.24	25.74 ± 17.9	*p* < 0.001

**Table 4:** Nipple-areolar complex (NAC) sensitivity and scar assessment.

NAC Sensitivity Assessment	
Monofilament	3.07 ± 1.3
Discrimination	16.84 ± 6.06
Light touch	2.65 ± 1.29
Vibration	2.78 ± 1.03
Cold	2.37 ± 1.46
Average Vancouver scar scale score (0–13);	1.79 ± 0.98

**Table 5:** BREAST-Q score and time of follow-up (months) correlation.

	N	Pearson Correlation	Sig. (2-Tailed)
Satisfaction with Chest (11–44)	20	0.022	0.926
Satisfaction with Outcome (5–15)	20	−0.473	0.035
Psychosocial Well-Being (9–45)	20	0.206	0.383
Sexual Well-Being (6–30)	20	0.039	0.890
Physical Well-Being (0–32)	20	−0.296	0.204
Satisfaction with NAC appearance (5–20)	20	−0.107	0.662
Satisfaction with Information (10–40)	20	−0.165	0.501
Satisfaction with Surgeon (12–48)	20	0.143	0.549
Satisfaction with Medical Team (7–28)	20	−0.304	0.192
Satisfaction with Office Staff (7–28)	20	−0.105	0.658

**Table 6:** Correlations between various domains of the BREAST Q questionnaires.

	Satisfaction with Chest	Satisfaction with Outcome	Psychosocial Well-Being	Sexual Well-Being	Physical Well-Being	Satisfaction with NAC appearance	Satisfaction with Information	Satisfaction with Surgeon	Satisfaction with Medical Team	Satisfaction with Office Staff
Satisfaction with Chest	1	R = 0.31 *p* = 0.18	R = 0.662 *p* = 0.01	R = 0.49 *p* = 0.06	R = −0.04 *p* = 0.85	R = 0.603 *p* = 0.006	R = 0.717 *p* < 0.001	R = 0.27 *p* = 0.26	R = 0.531 *p* = 0.016	R = 0.525 *p* = 0.018
Satisfaction with Outcome	R = 0.30 *p* = 0.18	1	R = 0.27 *p* = 0.24	R = 0.50 *p* = 0.057	R = 0.39 *p* = 0.083	R = 0.22 *p* = 0.35	R = 0.481 *p* = 0.037	R = −0.019 *p* = 0.94	R = 0.239 *p* = 0.31	R = 0.22 *p* = 0.34
Psycho-social Well-Being	R = 0.662 *p* = 0.001	R = 0.274 *p* = 0.24	1	R = 0.528 *p* = 0.043	R = 0.21 *p* = 0.37	R = 0.41 *p* = 0.08	R = 0.49 *p* = 0.034	R = 0.45 *p* = 0.044	R = 0.28 *p* = 0.24	R = 0.52 *p* = 0.019
Sexual Well-Being	R = 0.496 *p* = 0.06	R = 0.50 *p* = 0.057	R = 0.528 *p* = 0.043	1	R = 0.26 *p* = 0.35	R = −0.12 *p* = 0.68	R = 0.44 *p* = 0.12	R = 0.27 *p* = 0.32	R = 0.24 *p* = 0.38	R = 0.39 *p* = 0.15
Physical Well-Being	R = −0.04 *p* = 0.85	R = 0.39 *p* = 0.083	R = 0.21 *p* = 0.37	R = 0.26 *p* = 0.35	1	R = 0.18 *p* = 0.45	R = −0.07 *p* = 0.78	R = 0.10 *p* = 0.66	R = −0.09 *p* = 0.69	R = −0.10 *p* = 0.66
Satisfaction with NAC appearance	R = 0.60 *p* = 0.006	R = 0.23 *p* = 0.35	R = 0.41 *p* = 0.082	R = −0.12 *p* = 0.68	R = 0.18 *p* = 0.45	1	R = 0.38 *p* = 0.11	R = 0.16 *p* = 0.52	R = 0.45 *p* = 0.054	R = 0.29 *p* = 0.22
Satisfaction with Information	R = 0.717 *p* < 0.001	R = 0.481 *p* = 0.037	R = 0.489 *p* = 0.034	R = 0.44 *p* = 0.12	R = 0.07 *p* = 0.78	R = 0.38 *p* = 0.11	1	R = 0.486 *p* = 0.035	R = 0.62 *p* = 0.005	R = 0.65 *p* = 0.003
Satisfaction with Surgeon	R = 0.27 *p* = 0.26	R = −0.02 *p* = 0.94	R = 0.454 *p* = 0.044	R = 0.27 *p* = 0.32	R = −0.10 *p* = 0.66	R = 0.16 *p* = 0.52	R = 0.486 *p* = 0.035	1	R = 0.41 *p* = 0.069	R = 0.755 *p* < 0.001
Satisfaction with Medical Team	R = 0.531 *p* = 0.016	R = 0.24 *p* = 0.31	R = 0.28 *p* = 0.24	R = 0.24 *p* = 0.38	R = −0.09 *p* = 0.69	R = 0.449 *p* = 0.054	R = 0.62 *p* = 0.005	R = 0.41 *p* = 0.069	1	R = 0.818 *p* < 0.001
Satisfaction with Office Staff	R = 0.525 *p* = 0.018	R = 0.22 *p* = 0.34	R = 0.52 *p* = 0.019	R = 0.39 *p* = 0.15	R = 0.10 *p* = 0.66	R = 0.29 *p* = 0.22	R = 0.650 *p* = 0.003	R = 0.755 *p* < 0.001	R = 0.818 *p* < 0.001	1

**Table 7:** BREAST-Q score and monofilament NAC sensitivity assessment correlation.

	N	Pearson Correlation	Sig. (2-Tailed)
Satisfaction with Chest (11–44)	19	0.222	0.361
Satisfaction with Outcome (5–15)	19	0.076	0.758
Psychosocial Well-Being (9–45)	19	0.532	0.019
Sexual Well-Being (6–30)	19	−0.229	0.432
Physical Well-Being (0–32)	19	0.076	0.757
Satisfaction with NAC appearance (5–20)	19	0.398	0.102
Satisfaction with Information (10–40)	19	0.288	0.247
Satisfaction with Surgeon (12–48)	19	0.418	0.075
Satisfaction with Medical Team (7–28)	19	0.389	0.099
Satisfaction with Office Staff (7–28)	19	0.393	0.096

## Data Availability

The data that support the findings of this study are available from the corresponding author upon reasonable request.
